# Inhibition of NNMT enhances drug sensitivity in lung cancer cells through mediation of autophagy

**DOI:** 10.3389/fphar.2024.1415310

**Published:** 2024-07-05

**Authors:** Jian Wang, Ming Zhang, Xin You, Yang Xu, Congcong Zhang, Ying Li, Chunhui Yang, Qi Wang

**Affiliations:** ^1^ Translational Research Center for Lung Cancer, The Second Hospital, Dalian Medical University, Dalian, Liaoning, China; ^2^ Department of Respiratory Medicine, The Second Hospital, Dalian Medical University, Dalian, Liaoning, China; ^3^ Department of Respiratory Medicine, Affiliated Zhongshan Hospital of Dalian University, Dalian, China; ^4^ Department of Clinical Laboratory, The Second Hospital, Dalian Medical University, Dalian, Liaoning, China

**Keywords:** NNMT, drug sensitivity, autophagy, machine learning, osimertinib

## Abstract

**Introduction:**

This study aimed to investigate the role of Nicotinamide N-methyltransferase (NNMT) in the drug sensitivity of non-small cell lung cancer (NSCLC) cells, with a focus on its impact on autophagy and resistance to the chemotherapeutic agent osimertinib. The study hypothesized that NNMT knockdown would enhance drug sensitivity by modifying autophagic processes, providing a potential new therapeutic target for overcoming chemoresistance in lung cancer.

**Methods:**

Proteomic analysis was utilized to identify changes in protein expression following NNMT knockdown in H1975 and H1975 osimertinib resistance (H1975OR) lung cancer cell lines. Gene expression patterns and their correlation with NNMT expression in lung cancer patients were analyzed using The Cancer Genome Atlas (TCGA) dataset. Additionally, a predictive model for lung cancer survival was developed via lasso regression analysis based on NNMT-associated gene expression. Drug sensitivity was assessed using the IC50 values and apoptosis ratio, and autophagy was evaluated through Western blot and flow cytometric analysis.

**Results:**

Significant variations in the expression of 1,182 proteins were observed following NNMT knockdown, with a significant association with autophagy-related genes. Analysis of gene expression patterns unveiled a significant correlation between NNMT expression and specific changes in gene expression in lung cancer. The predictive model successfully forecasted lung cancer patient survival outcomes, highlighting the potential of NNMT-associated genes in predicting patient survival. Knockdown of NNMT reversed osimertinib resistance in H1975 cells, as evidenced by altered IC50 values and apoptosis ratio, and changes were observed in autophagy markers.

**Discussion:**

Knockdown of NNMT in lung cancer cells enhances drug sensitivity by modulating autophagy, providing a promising therapeutic target to overcome chemoresistance in NSCLC. The study underscores the importance of NNMT in lung cancer pathology and underscores its potential as a predictive marker for clinical outcomes. Additionally, the developed predictive model further supports the clinical relevance of NNMT-associated gene expression in improving the prognosis of lung cancer patients.

## 1 Introduction

Lung cancer, predominantly NSCLC, stands as a leading cause of cancer-related mortality globally (Campbell et al., 2019). Despite advancements in targeted therapies and immunotherapy (Lovly and Horn, 2012; Wang et al., 2021; Guler et al., 2024), the prognosis for lung cancer patients, especially those with advanced-stage disease, often remains modest (Rivera-Concepcion et al., 2022). A major challenge in the treatment of lung cancer is the development of resistance to chemotherapeutic agents, underscoring the need for new therapeutic approaches and targets. Among various potential targets, metabolic enzymes like NNMT have drawn attention because of their roles in cancer progression (Arefnezhad et al., 2024; Huang et al., 2024; Ma et al., 2024). NNMT, involved in the methylation of nicotinamide and other pyridines, has been linked to cancer cell metabolism, growth, and metastasis. Ma et al. found that vanillin can downregulate NNMT and attenuate NNMT-related resistance to 5-fluorouracil via ROS-induced cell apoptosis in colorectal cancer cells (Li et al., 2021). Nicotinamide N-methyltransferase gene silencing enhances the chemosensitivity of melanoma cell lines (Campagna et al., 2021). Importantly, emerging evidence suggests an inverse relationship between NNMT expression and chemoresistance in lung cancer (Bach et al., 2018). A crucial process relevant to cancer biology and treatment response is autophagy, which is a cellular mechanism for the degradation and recycling of cellular components (Kocaturk et al., 2019). Autophagy plays a dual role in cancer; it can suppress tumor formation by removing damaged organelles and proteins but can also promote cancer cell survival under stress conditions, such as exposure to chemotherapeutic drugs (Yun and Lee, 2018). The intricate role of autophagy in mediating drug resistance is particularly noteworthy in lung cancer, and understanding this relationship is critical for developing more effective treatments (Tonkin-Reeves et al., 2023). Preliminary data suggest an inverse relationship between NNMT expression and autophagy in lung cancer cell lines, forming the basis of our hypothesis. By investigating the interplay between NNMT, autophagy, and drug sensitivity, our aim is to identify a novel therapeutic target that could potentially enhance the efficacy of lung cancer treatments.

## 2 Materials and methods

### 2.1 Access and analysis of public data

The whole-genome expression profiles and clinicopathological information of human cancer were obtained from The Cancer Genome Atlas (TCGA) (https://tcga-data.nci.nih.gov/). To compare lung cancer tumor tissue samples, an unpaired *t*-test was employed. The screening criteria were upregulated or downregulated log2FC ≥ 2.0 and FDR <0.05. Survival analysis was performed using the survival package in R (version 3.5.4) to evaluate patients’ survival times. The log-rank test was utilized to examine variations in survival rates across various patient groups. To handle high-dimensional data and select significant predictors, we employed lasso analysis through the glmnet package in R. Heatmaps were generated using the pheatmap package, facilitating the intuitive presentation of gene expression data and patterns. The generation of patient survival curves and the analysis of gene associations were conducted via the ENCORI/starBase (https://rnasysu.com/encori/) websites.

### 2.2 Cell culture

H1975 and H1975OR cells were obtained from Guangzhou Medical University. The cells were cultured in RPMI 1640 medium (Dalian Meilunbio) supplemented with 10% fetal bovine serum (Gibco) and 1% penicillin/streptomycin (Dalian Meilunbio), and maintained at 37°C in a 5% CO_2_ atmosphere. Passage was conducted using 0.25% trypsin-EDTA solution containing PhenolRed (Dalian Meilunbio).

### 2.3 Construction of stable transfected cell lines

The stable transfection virus solution was obtained from GENECHEM (GIEL0344657). H1975OR cells were plated at a density of 2 × 10^5^ cells per well in 6-well plates, and the appropriate amount of virus solution was calculated based on the MOI = 20 instruction. The complete medium was replaced 16 h after adding the viral solution and pro-transfection agent. Stable transfected cell lines were established after 5 days of continuous puromycin (0.1 mg/mL, MCE) selection. The target sequence of NNMT shRNA was as follows: ACC​CTC​GGG​ATT​ACC​TAG​AAA, GCT​CAA​GAG​CAG​CTA​CTA​CAT, GTG​ACC​TAT​GTG​TGT​GAT​CTT.

### 2.4 Cycle flow assay

Harvest 4 × 10^5^ cells into a centrifuge tube and centrifuge at 300 *g* for 5 min to remove the culture medium. Wash the cells once with 1 mL of cold PBS, centrifuge again, and discard the supernatant. Resuspend the cell pellet in approximately 1 mL of anhydrous ethanol pre-cooled at −20°C, gently vortex to mix, and fix overnight at −20°C. Afterward, wash the cells once with 1 mL of cold PBS (pre-cooled at 4°C), centrifuge at 300 *g* for 10 min, and discard the supernatant. Repeat the centrifugation step at 300 g for 5 min, aspirate the supernatant, and resuspend the cells in 100 μL of RNase A. Incubate the cells at 37°C. Wash the cells once with 1 mL of cold PBS (pre-cooled at 4°C), centrifuge at 300 *g* for 10 min, and discard the supernatant. Afterward, centrifuge again at 300 g for 5 min, aspirate the supernatant, and add 100 μL of RNase A to fully suspend the cells. Incubate the cells in a water bath at 37°C for 30 min. Add 400 μL of propidium iodide (PI) solution to the cells and mix thoroughly. Incubate the cells for 30 min at 4°C, protected from light (using the Annexin V-FITC/PI Apoptosis Kit). Subsequently, analyze red fluorescence at an excitation wavelength of 488 nm using flow cytometry. Acquire the cells at low speed and analyze DNA content using analysis software.

### 2.5 Apoptosis detection

Collect 4 × 10^5^ cells in a centrifuge tube, centrifuge at 300 *g* for 5 min, remove the culture medium; resuspend cells in PBS. Centrifuge at 300 *g* for 5 min, discard the supernatant. Resuspend cells in PBS, centrifuge at 300 g for 5 min, discard the supernatant. Resuspend the cells in 100 μL of the physiological buffer (1×), and add 2.5 μL each of Annexin V and PI (Cell Cycle Assay Kit). Incubate at room temperature in the dark for 15–20 min, then add 400 μL of the physiological buffer (1×). Detect the red fluorescence at the excitation wavelength of 488 nm using a flow cytometer, and acquire the cells at medium speed.

### 2.6 DIPI nuclear staining

Prepare the cells for the experiment by rinsing them thrice with PBS for 10 min. Perform paraformaldehyde fixation for 10 min. Rinse the cells again thrice with PBS for 10 min, then stain with 100 μL of DIPI for 20 min. Rinse the cells thrice with PBS for 10 min and observe under a fluorescence microscope with an excitation wavelength of 359 nm. Count the number of apoptotic cells.

### 2.7 Western blot

Harvest cells in a lysis buffer containing a mixture of protease inhibitors (Selleck). Following protein concentration assessment using the BCA protein detection kit (Dalian Meilunbio), equal amounts of denatured proteins were subjected to SDS-PAGE electrophoresis. The proteins were then transferred to a polyvinylidene difluoride membrane. The membranes were then sealed, incubated with primary and secondary antibodies, and subsequently visualised using a chemiluminescence imager. We cleaned the bolt using protein blotting regeneration solution (Dalian Meilunbio) and re-incubated the primary antibody secondary antibody. Primary antibodies against the following proteins were used: NNMT (15123-1-AP, ProteinTech), GAPDH (60004-1-Ig, ProteinTech), p62 (80294-1-RR, ProteinTech), LC3 (81004-1-RR, ProteinTech), C-Parp (9544T, Cell Signaling Technology), C-cas3 (9661T, Cell Signaling Technology), Bax (60627-1-IP, ProteinTech), Bcl-2 (12789-1-AP, ProteinTech), CDK4 (11026-1-AP, ProteinTech), Cyclin B1 (4138, Cell Signaling Technology). The blots were subsequently visualised using ImageJ software and GraphPad Prism for processing and analysis.

### 2.8 CCK-8 cell proliferation assay

Distribute 100 μL of cell suspension containing 4000 cells evenly across 96-well plates. After allowing the cells to adhere for 24 h, appropriate drugs were added to each well according to the respective groups, and the drugs were diluted in the control group. Prepare the reagents according to the CCK8 kit: Dilute the reagents in the working solution with normal medium at a ratio of 1:9. The working solution should be diluted 1:9 with normal medium. After incubation at 37°C for 2 h, add 100 μL of the working solution to each well, protecting from light. Measure the absorbance at 450 nm using an enzyme marker, and then process and analyze the results using Excel and GraphPad Prism.

### 2.9 Proteomic analysis

Trypsin was obtained from Sigma (St. Louis, MO). Chemical reagents were obtained from Sigma (St. Louis, MO). Formic acid (FA) was purchased from Fluka (Buches, Germany). Acetonitrile and water for RPLC (HPLC grade) was purchased from Merck (Darmstadt, Germany). Urea was obtained from Bio Basic Inc. (Ontario, Canada). Pure water for protein digestions and peptide enrichment was purified with a Milli-Q system (Millipore, Milford, MA). The H1975 and H1975OR cell lines were harvested by adding cell lysis buffer (6 M Gua, 50 mM HEPES, 40 mM CAA, 10 mM TCEP, pH = 7.4). Then the cell lysate solution was incubated at 95°C for 5 min. The following ultrasonication step were performed by Bioruptor for 20 min. After incubation at 95°C for 5 min, the protein solution was laid on ice for the following protein purification steps. The concentration of extracted proteins was measured by BCA kits (Beyotime, China). The protein solution containing 100 μg amount of protein for each sample were added to the ultrafiltration kits (Sartorius, Germany) for purification. Each of the protein solution was washed three times by 50 mM NH_4_HCO_3_ (pH = 7.4) and digested with trypsin at 37°C overnight. After digestion, the peptides from H1975 and H1975OR were collected by centrifugation and lyophilized to dryness. The obtained peptides were kept at −80°C until LC-MS/MS analysis. The analysis of peptides from H1975 and H1975OR cell lines was performed by using Q-Exactive 480 mass spectrometer equipped with a U3000 RSLC (Thermo, San Jose, CA, USA). To ensure the amount of loading peptides from each sample was consistent, the concentration of sample solution was validated by NanoDrop (Thermo, USA). The peptides were loaded and separated by a commercial column (AC-QUITY UPLC Peptide CSH C18 Column, Waters). The analysis gradient was as follows: 0–1 min, 4%–6% Buffer B (80%ACN/20%H_2_O/0.1%FA); 1–80 min, 6%–32% Buffer B; 80–93 min, 32%–45% Buffer B; 93–94 min, 45%–90% Buffer B; 93–98 min, 90% Buffer B; 98–100 min, 90%–4% Buffer B. The MS analysis was performed at DIA (data independent acquisition) mode with scan range (m/z) of 350–1,400 at resolution of 120,000. The maximum injection time was set as 45 ms and the normalized collision energy (NCE) was set as 30%. The *. Raw files obtained from MS were searched against uniprot database (20,618 sequences, 2022, www.uniprot.org) with Spectronaut software. The parameters were as follows: enzyme specificity was set to KR/P with up to two missed sites; cysteine residue was set as a static modification of 57.0215 Da; methionine oxidation (+15.9949 Da) were set as variable modifications. And the rest of the parameters were set as defaults. The quantification mode at peptide level was performed by “local normalization” strategy. The quantification results were further processed with Perseus software (www.maxquant.org/perseus) to obtain more quantification information. A total of 5,329 proteins were identified. Out of these, 1,182 proteins exhibited significant variation, with -Lg *p*-value >2.

### 2.10 Statistical analysis

Heatmap was plotted by https://www.bioinformatics.com.cn (last accessed on 20 February 2024), an online platform for data analysis and visualization. Statistical analysis was conducted with data represented as mean ± SD from a minimum of three separate experiments. ImageJ 1.54i was used for image analysis, and GraphPad Prism 9.0 was utilized for all data assessments. Comparisons between two experimental groups were made using an unpaired *t*-test. *p*-value of less than 0.05 was deemed to indicate statistical significance.

## 3 Results

### 3.1 Proteomic analysis reveals significant differences in protein expression and association with autophagy in H1975OR cells

**FIGURE 1:**
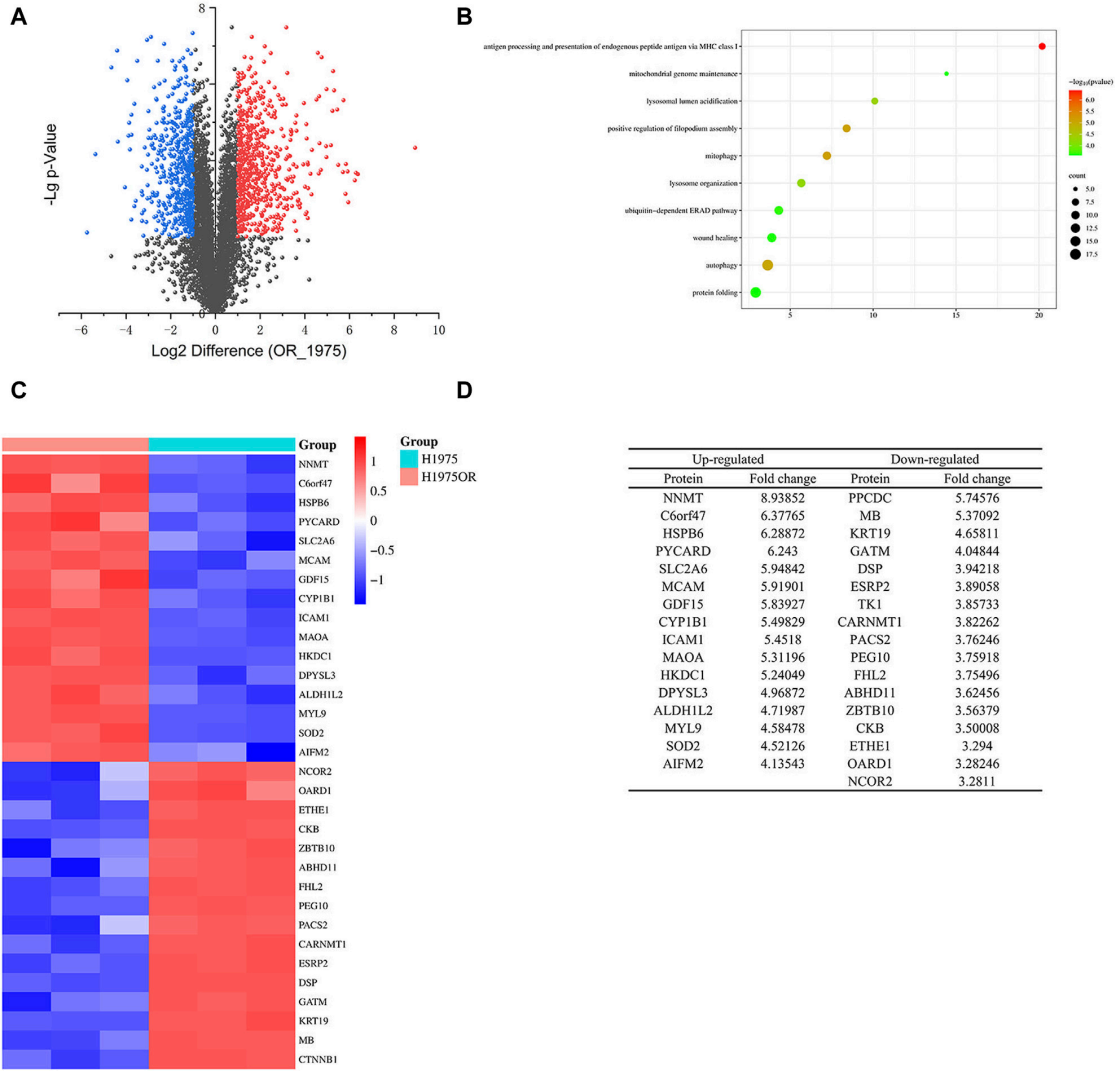
**(A)** The differential proteins of H1975 and H1975OR cells. Blue for proteins downregulated in H1975OR with -Lg *p*-value >2; Red for proteins upregulated in H1975OR with -Lg *p*-value >2; **(B)** The GO and KEGG enrichment analysis of the genes coding for these proteins. **(C,D)** Heatmap and table of differentiated proteins in H1975 and H1975OR, examples of 32 proteins that were significantly different.

In this study, we conducted proteomic analysis on H1975 and H1975OR cells, resulting in the screening of 5,329 proteins. Out of these, 1,182 proteins exhibited significant variation, with -Lg *p*-value >2 (Figures 1A,C,D). Among the significantly altered proteins, 640 exhibited higher expression levels in H1975OR cells compared to 542 proteins that showed lower expression levels. The protein NNMT stood out as the most significantly altered in expression. Furthermore, a comprehensive analysis of the genes coding for these proteins using gene ontology (GO) and Kyoto Encyclopedia of Genes and Genomes (KEGG) enrichment revealed a significant association with autophagy-related genes in H1975OR cells (Figure 1B).

### 3.2 NNMT expression and its correlation with gene expression patterns in lung cancer

**FIGURE 2:**
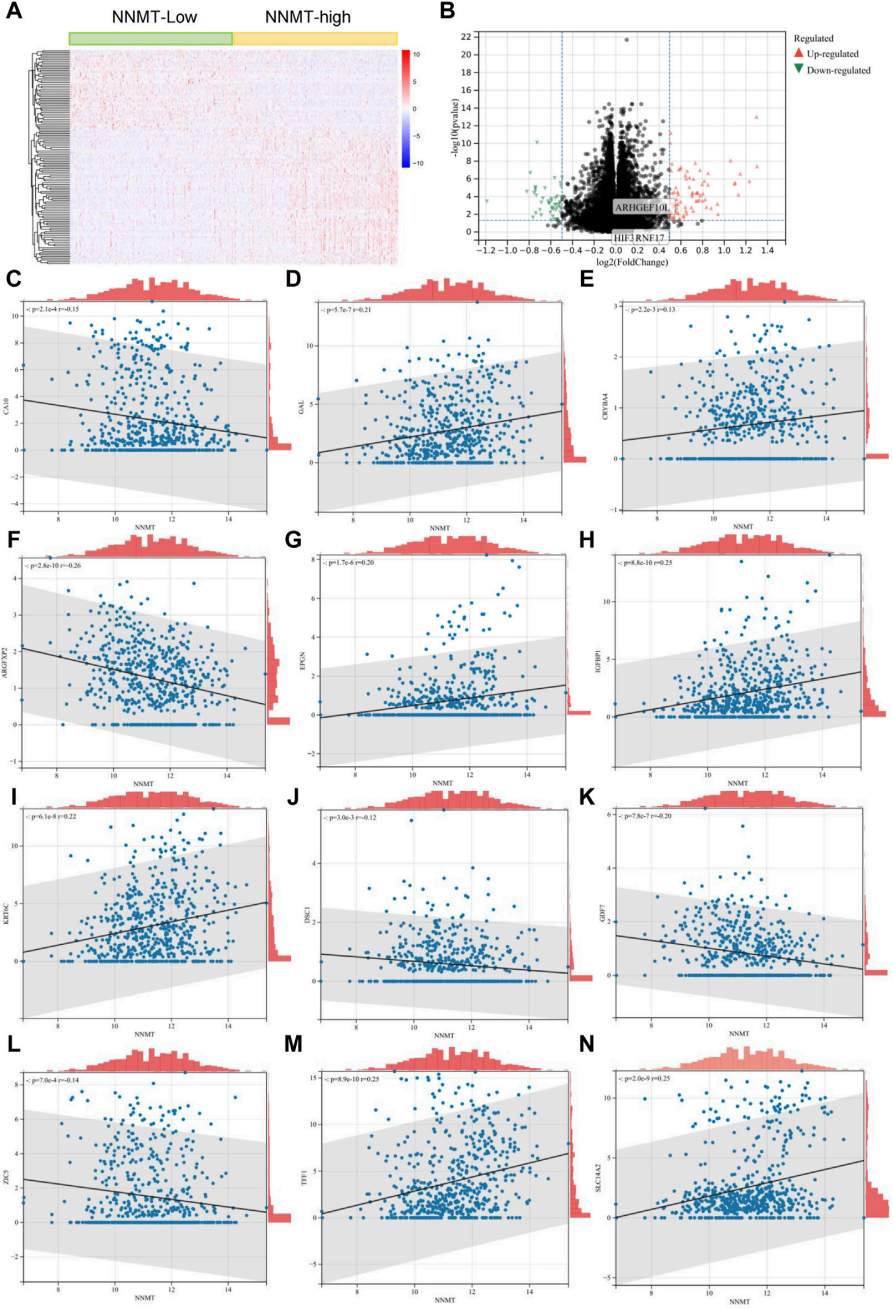
NNMT Expression and Its Correlation with Gene Expression Patterns in Lung Cancer. **(A)** Heatmap depicting differential gene expression between high and low NNMT expression groups in lung cancer patients from the TCGA dataset. Each row represents a gene, and each column represents a patient. Color intensity reflects gene expression levels, with red indicating higher expression and blue indicating lower expression. **(B)** Volcano plot showing the significance versus fold-change of gene expression differences between high and low NNMT expression groups. Points represent individual genes; red points indicate significantly upregulated genes, green points indicate significantly downregulated genes, and black points represent genes with no significant difference in expression. **(C–N)** These analyses reveal significant correlations, indicating that NNMT expression might be closely associated with specific gene expression changes in lung cancer, suggesting its potential role in the disease’s pathology and as a marker for clinical outcomes. These genes include: CA10, GAL, CRYBA4, ARGFXP2, IGFNP1, KRT6C, GDF7, ZIC5, SLC14A2, EPGN, DSC1 and TFF1.

In our analysis, we classified TCGA lung cancer patients into two groups based on the expression levels of the NNMT gene, distinguishing them between high and low expression. The differential gene expression between these groups is shown in Figure 2A, a heatmap that highlights the significant variations in gene expression patterns. Figures 2A, B volcano plot, further illustrates these differences, showing genes significantly upregulated or downregulated in relation to NNMT expression levels. Subsequent correlation analyses detail the relationships between the expression of NNMT and the differentially expressed genes. These genes include: CA10, GAL, CRYBA4, ARGFXP2, IGFNP1, KRT6C, GDF7, ZIC5, SLC14A2, EPGN, DSC1 and TFF1. These genes are related to cell growth, metabolism, and signal transduction. These analyses reveal significant correlations, indicating that NNMT expression may be closely linked with specific gene expression changes in lung cancer, suggesting its potential role in the pathology of the disease and as a marker for clinical outcomes (Figures 2C–N).

### 3.3 Development of a predictive model for lung cancer survival based on NNMT-associated gene expression

**FIGURE 3:**
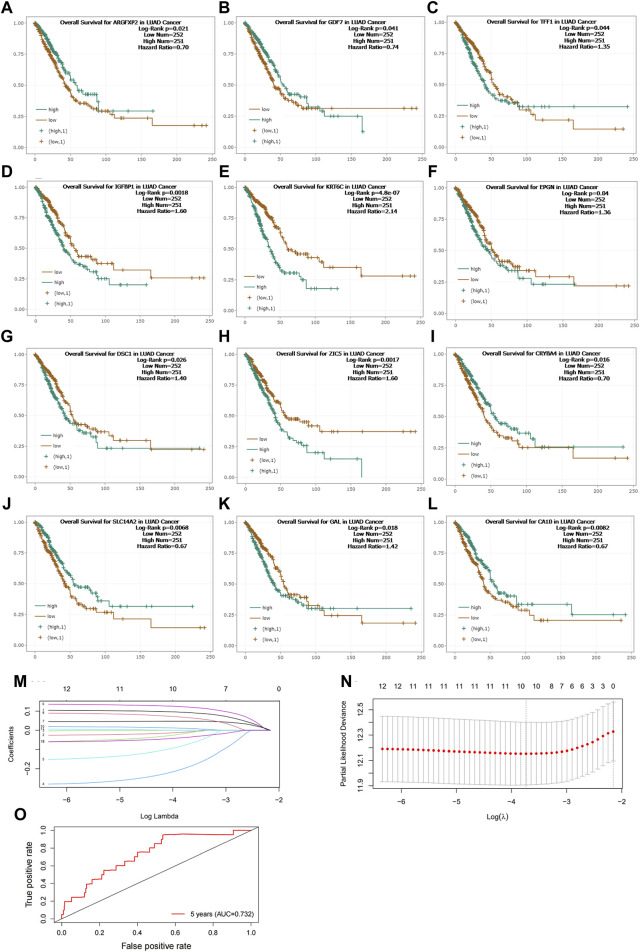
**(A–L)** Survival Analysis of Lung Cancer Patients Based on NNMT-Associated Gene Expression. This study explored the correlation between NNMT and other genes in lung cancer. Patients were divided into high and low expression groups for these genes based on median gene expression levels, and survival analyses were conducted. **(M,N)** Predictive Model for Lung Cancer Patient Survival Outcomes Constructed Using Lasso Regression Analysis. The model refined to include parameters for 10 genes. **(O)** The ROC of Predictive Model. Risk Score = IGFBP1 × 0.0843 - CA10 × 0.0189 - CRYBA4 × 0.2311 - ARGFXP2 × 0.1468 + EPGN × 0.1024 + KRT6C × 0.0475 + DSC1 × 0.0777 - GDF7 × 0.0373 + ZIC5 × 0.0173 - SLC14A2 × 0.0498. The ROC of the Predictive Model is 0.732.

In our study, we investigated the correlation between NNMT and other genes in lung cancer. Utilizing the median gene expression levels, we divided patients into high and low expression groups for these genes and performed survival analyses. Our findings revealed that 12 genes exhibited significant differences in survival outcomes, underscoring the potential of NNMT-associated genes to predict patient survival (Figures 3A–L). Further, we employed lasso regression analysis to integrate the expression of these correlated genes with patient survival times, aiming to construct a predictive model. The lasso regression, which is known for its ability to perform variable selection and regularization, aided in refining our model to include the most predictive features. Ultimately, our model incorporated parameters for 10 genes, effectively predicting lung cancer patient survival outcomes (Figures 3M, N). The risk scoring formula was as follows: Risk Score = IGFBP1 × 0.0843 - CA10 × 0.0189 - CRYBA4 × 0.2311 - ARGFXP2 × 0.1468 + EPGN × 0.1024 + KRT6C × 0.0475 + DSC1 × 0.0777 - GDF7 × 0.0373 + ZIC5 × 0.0173 - SLC14A2 × 0.0498. The ROC of the Predictive Model is 0.732 (Figure 3O).

### 3.4 Knockdown of NNMT enhances drug sensitivity through mediation of autophagy

**FIGURE 4:**
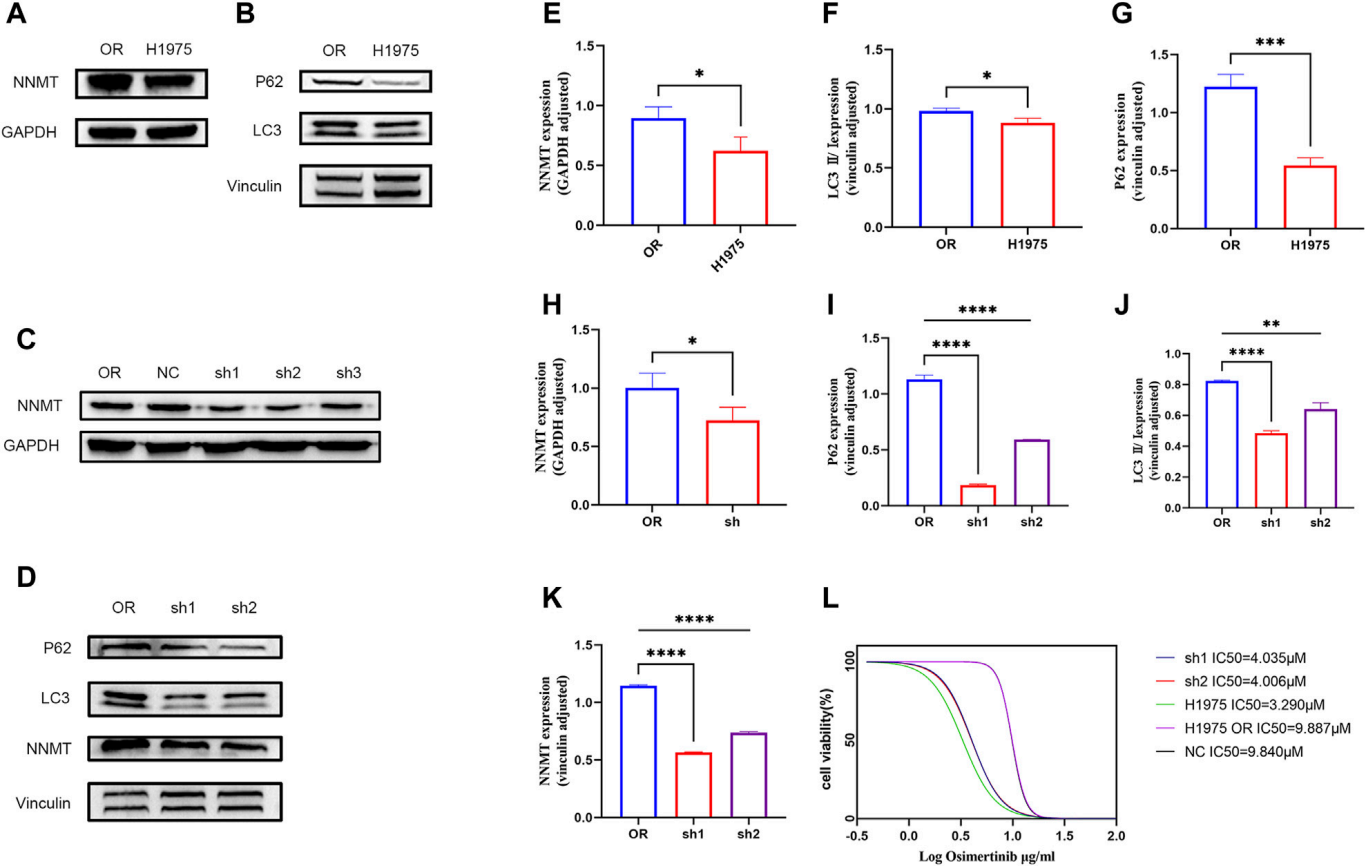
**(A,B)** Differences in the expression of NNMT and autophagy between H1975 and H1975OR cells. **(C)** Construction of NNMT knockdown stable-turnover cells. **(D)** Differences in the expression of NNMT and autophagy between H1975OR, sh1 and sh2. **(E–K)** Statistical analysis of protein expression differences (*p*-value <0.05). Quantitation of these results, with comparison using an unpaired two-tailed *t*-test. **(L)** The IC50 value of H1975, H1975OR, NC, sh1, sh2. *(*p* < 0.05), **(*p* < 0.01), ***(*p* < 0.001), and ****(*p* < 0.0001).

Based on proteomic findings, we observed that NNMT was highly expressed in the H1975OR cell line and this was confirmed in Western blot (Figures 4A, E). NNMT is usually accompanied by altered autophagy, and our results indicated that autophagic flux was reduced in H1975OR cells (Figures 4B, F, G). To verify whether NNMT is associated with osimertinib resistance, we generated stable NNMT knockdown cell lines (Figures 4C, H). We found that NNMT knockdown reversed drug resistance. The IC50 of sh1 and sh2 was significantly different from that observed in H1975OR cells. The IC50 value of H1975OR is 9.887 μM. The sh1 and sh2 are 4.035 μM and 4.006 μM (Figure 4L). NNMT is commonly associated with autophagy, and our results showed that knockdown of NNMT increased autophagy (Figures 4D, I, J, K). Therefore, NNMT may be reversing lung cancer resistance to osimertinib through mediating autophagy.

### 3.5 NNMT affects cell proliferation and apoptosis through mediation of autophagy

**FIGURE 5:**
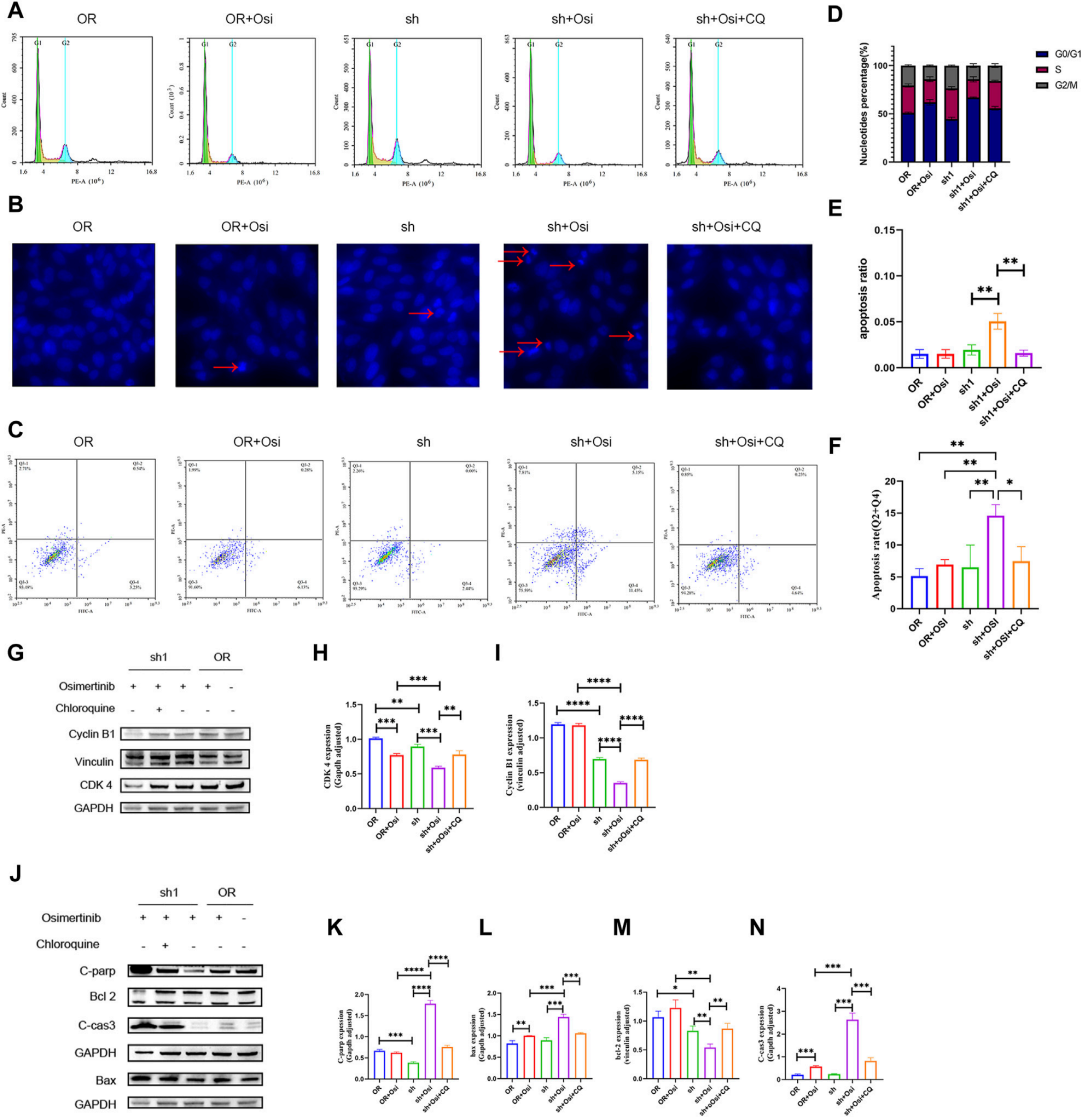
The detection of cell cycle and apoptosis between H1975OR, OR + Osimertinib, sh, sh + Osimertinib and sh + Osimertinib + Chloroquine. The osimertinib concentration was 4 μM for 24 h of action and Chloroquine concentration was 20 μM for 24 h of action. **(A)** Cell cycle assay **(B)** DIPI nuclear staining assay. Using fluorescence microscope ingestion, 10 images were collected per field of view and the percentage of total cells in the presence of early apoptotic and late apoptotic cells was calculated. **(C)** Flow detection of apoptosis. **(D–F)** Statistical analysis of cell cycle, DIPI nuclear of apoptosis and flow detection of apoptosis (Q3-2 + Q3-4). Quantitation of these results, with comparison using an unpaired two-tailed *t*-test. **(G,J)** Differences in the expression of cycle and apoptosis between H1975OR, OR + Osimertinib, sh, sh + Osimertinib and sh + Osimertinib + CQ. CDK4 and Bax in the same bolt. **(H,I)** and **(K–N)** Statistical analysis of protein expression differences. *(*p* < 0.05), **(*p* < 0.01), ***(*p* < 0.001), and ****(*p* < 0.0001).

Knockdown of NNMT resulted in significant cell cycle alterations, primarily characterized by G2/M changes. H1975OR and sh1 cells significantly lagged in G2/M after the addition of osimertinib, indicating that osimertinib affected the G2/M phase of the cells. Knockdown of NNMT resulted in increased expression of autophagy in cells, and therefore, we added chloroquine to the dosing group and found that inhibition of autophagy reversed the cell cycle changes (Figures 5A, D). Concurrently, it was observed that the expression of cyclin B1 and CDK4 decreased following the knockdown of NNMT. The decrease in expression was reversed by chloroquine (Figures 5G–I). We added osimertinib to NNMT knockdown cells. This resulted in significant apoptotic vesicles and nuclear division, as confirmed by a flow assay (Figures 5B, C, E, F). Additionally, the levels of apoptotic proteins C-parp, bax, and c-cas3 were significantly elevated, whereas the anti-apoptotic protein Bcl-2 was decreased. Meanwhile, we found that autophagy inhibitors can reverse the trend towards apoptosis and the expression of related apoptotic proteins (Figures 5J–N).

## 4 Discussion

Autophagy, a highly conserved process in human evolution, degrades unwanted or damaged cytoplasmic contents in a lysosome-dependent manner (Shin et al., 2018). Differences in the type and stage of cancer pathology and the microenvironment allow autophagy to play different roles (Suzuki et al., 2020). In the early stages of tumorigenesis, autophagy suppresses tumorigenesis by eliminating damaged cells and maintaining cells in good condition. However, in the later stages, over-activated autophagy allows cancer cells to survive in a nutrient and oxygen-deficient environment, promoting tumorigenesis. Common autophagic targets associated with lung cancer include P53, the mTOR signaling pathway and endoplasmic reticulum stress (Liu et al., 2017). P53 can promote cell apoptosis and inhibit autophagy (Kruse and Gu, 2009). Patients with mutated EGFR often exhibit abnormal activation of the PI3K/AKT/mTOR pathway (Fumarola et al., 2014), leading to resistance to EGFR tyrosine kinase inhibitors (EGFR TKIs) in clinical treatment. mTORC1 and mTORC2 are two signaling complexes involved in the mTOR pathway. An important function of mTORC1 is to act as a negative regulator of autophagy (Loewith et al., 2002). Endoplasmic reticulum stress is also a key regulator of cell apoptosis and autophagy (Yadav et al., 2014). Studies have shown that reducing autophagy can increase the sensitivity of lung cancer cells to chemotherapy (Gewirtz, 2016). Rapamycin can inhibit mTOR activity, and the combination of rapamycin with the Bcl-2 inhibitor ABT-737 can increase apoptosis and autophagy *in vitro*, leading to better radiotherapy outcomes (Kim et al., 2009). While there are some conflicting reports on autophagy in lung cancer, the majority of studies suggest that inhibiting autophagy can enhance anti-tumour effects. The combination of autophagy inhibitors with chemotherapy has made significant advances in the treatment of lung cancer.

In this study, we found that NNMT contributes to NSCLC resistance to osimertinib through mediation of autophagy. Thus, the activity of autophagy varies with the environment. Epidermal growth factor receptor inhibitors such as gefitinib and ositinib have been found to induce autophagy in NSCLC cell lines. However, one of the mechanisms of acquired drug resistance in NSCLC is protective autophagy (Cao et al., 2022). Drug-resistant cells exhibit higher autophagic activity compared to parental sensitive cells. Therefore, the role of autophagy in tumor drug resistance is still unclear, and autophagy is a “double-edged sword” that can promote both cell death and cell survival. However, existing studies have shown that cellular stress, including elevated drug stress, increases autophagy activity. During autophagy, the formation of double-membrane autophagosomes engulfs damaged organelles, pathogens, cellular proteins, and macromolecules and transports them to lysosomes. Inhibition of autophagy has been reported to reverse NSCLC resistance to epidermal growth factor receptor-TKI. NNMT is a lysosomal enzyme that utilizes S-adenosyl-L-methionine (SAM) as a donor to produce N1-methylnicotinamide (MNAM) and S-adenosyl-L-homocysteine (SAH) (Pozzi et al., 2022). These enzymes catalyze the N-methylation reaction of nicotinamide (NAM) and are typically highly expressed in the liver. Upregulation of NNMT is closely associated with its ability to promote various cellular pathways and processes contributing to tumorigenesis and progression, including cell proliferation, migration, invasion, and resistance to chemotherapy (Ehebauer et al., 2020). NNMT also plays a crucial role in lipid metabolism, adipocyte differentiation, and potentially obesity-related regulatory pathways (Xu et al., 2022). It has been suggested that NNMT may regulate adipocyte function and lipid metabolism through its regulatory effects on autophagy and adipocyte-related genes. However, the relationship between NNMT and autophagy is multifaceted. On one hand, NNMT is positively regulated with autophagy, and knockdown of NNMT leads to an increase in autophagic activity, affecting the survival and growth of tumor cells (Shin et al., 2018). NNMT may promote tumor growth and survival by inhibiting autophagy. On the other hand, NNMT and autophagy are negatively regulated, and NNMT protects tumor cells from nutrient deprivation by negatively regulating autophagic processes (Ehebauer et al., 2020). In neurodegenerative diseases, NNMT reduces the catalytic ability of LCMT-1 by decreasing the concentration of SAM, leading to a decrease in the activity of LET-92/PP2A (Schmeisser and Parker, 2018). Reduced LET-92/PP2A activity fails to dephosphorylate NPRL-2, thereby inducing autophagy. Autophagy promotes drug resistance through glycolysis, ROS, and cell stemness (Yu et al., 2020). NNMT inhibits oxidative stress-induced autophagy in breast cancer cells through the ROS-mediated AMPK-ULK1 pathway and may protect breast cancer cells from oxidative stress by inhibiting autophagy (Verusingam et al., 2021). Simultaneous autophagy and glycolysis are associated with ositinib resistance (Tang et al., 2017). The energetic complementarity and dynamic balance between these two processes make the resistance process difficult to block; breaking the complementary relationship between them can effectively overcome resistance (Chen et al., 2021). Activation of autophagic flux induces resistance to EGFR-TKIs in NSCLC cells (Fleisher et al., 2019). Autophagy also promotes cell survival under cancer stress conditions (Yeon et al., 2023). Resistance to ositinib was associated with enhanced autophagy and stem cell-like properties in EGFR-mutant NSCLC cells. Combination therapy of EGFR-TKIs with autophagy inhibitors may be an effective strategy to ameliorate ositinib cytotoxicity (Chen et al., 2019; Li et al., 2019). Proteomic analysis revealed high expression of NNMT in H1975-resistant cells, accompanied by significantly enriched autophagy-related genes. Additionally, protein blotting experiments showed that increased NNMT expression in H1975-resistant cells was accompanied by decreased autophagy flux. Knockdown of NNMT effectively reversed osimertinib resistance in lung cancer, while NNMT knockdown cell lines exhibited increased autophagic flux. Both H1975OR cells and NNMT knockdown cell lines displayed alterations in their cell cycle after oxitinib treatment, primarily resulting in a G2/M block. We used chloroquine to inhibit autophagic flux and observed the reversal of cell death, as confirmed in apoptosis experiments. Therefore, NNMT could contribute to ositinib resistance in lung cancer by affecting autophagy. However, this study has several limitations. Firstly, it did not follow up on the validation of the screened genes. Secondly, it did not explore the correlation between NNMT and autophagy, as well as the factors by which autophagic homeostasis affects apoptosis. We have identified NNMT and autophagy as potential targets for overcoming osimertinib resistance in NSCLC.

## Data Availability

The datasets presented in this study can be found in online repositories. The names of the repository/repositories and accession number(s) can be found in the article/Supplementary Material.
