# Supplementary figures and images for: Inhibition of NNMT enhances drug sensitivity in lung cancer cells through mediation of autophagy
Source: Front Pharmacol. 2024 Jul 5;15:1415310. doi: 10.3389/fphar.2024.1415310 (PMC11257979; doi:10.3389/fphar.2024.1415310)

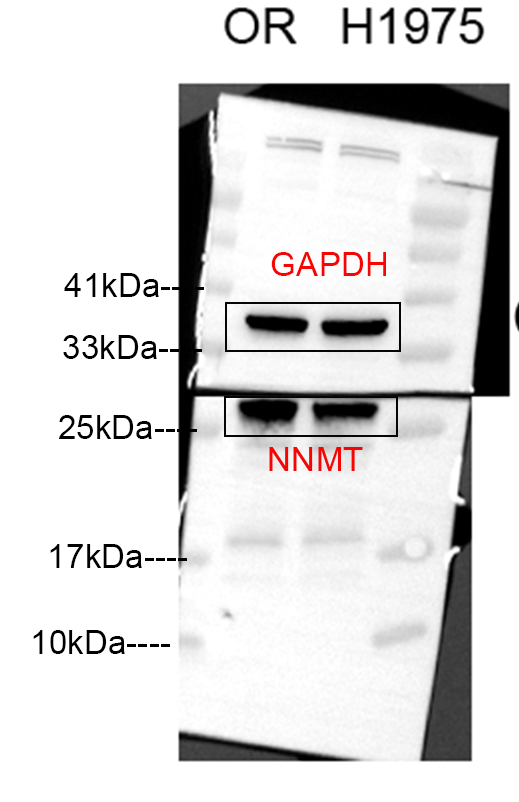

Supplement: Supplementary file 1 [file Image6.TIF]

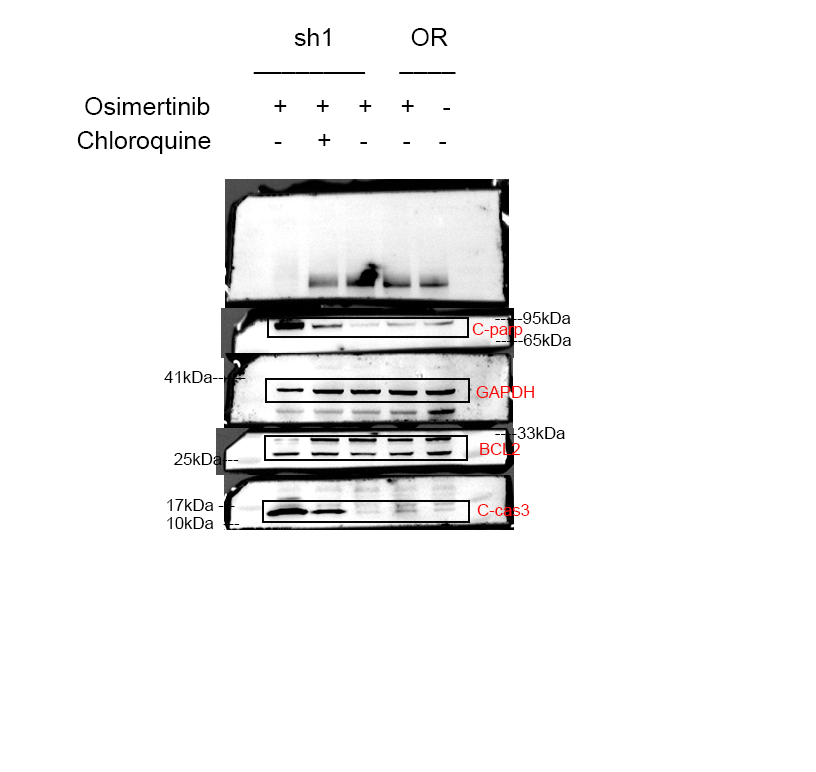

Supplement: Supplementary file 2 [file Image3.TIF]

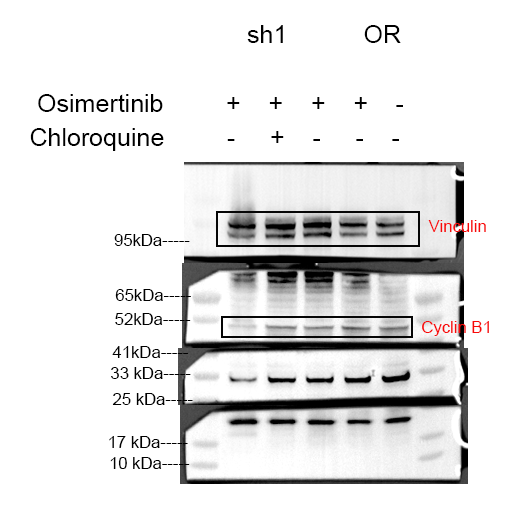

Supplement: Supplementary file 3 [file Image4.TIF]

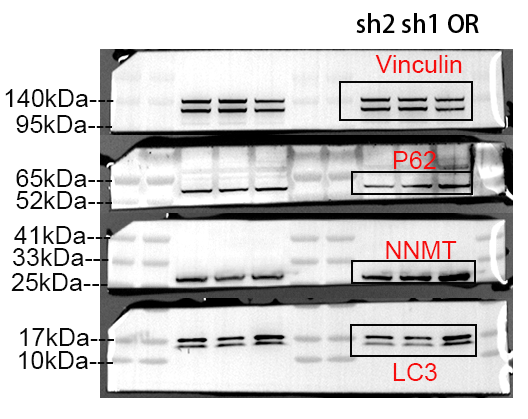

Supplement: Supplementary file 4 [file Image2.TIF]

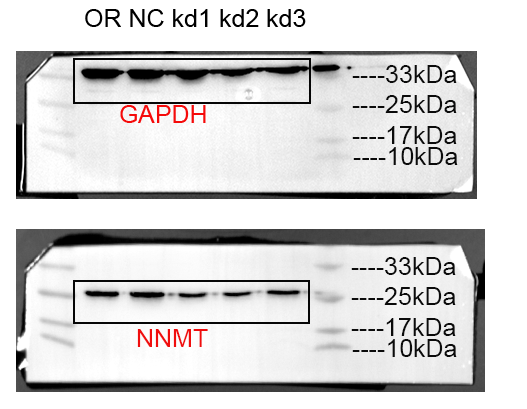

Supplement: Supplementary file 5 [file Image1.TIF]

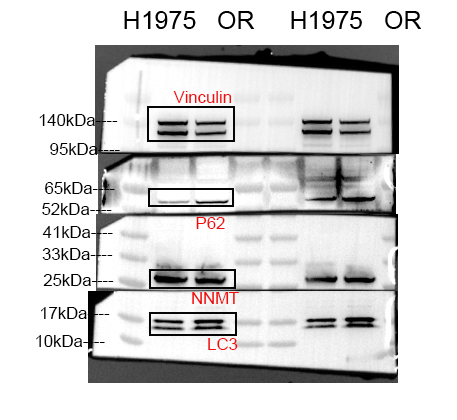

Supplement: Supplementary file 6 [file Image7.TIF]

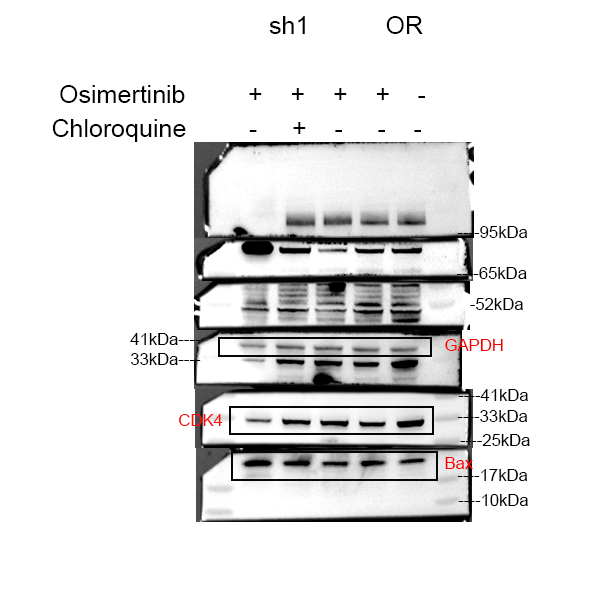

Supplement: Supplementary file 8 [file Image5.TIF]
